# Unilateral Abducens Nerve Palsy as the Lone Sign of Cerebral Venous Sinus Thrombosis: A Case Report and Literature Review

**DOI:** 10.7759/cureus.41350

**Published:** 2023-07-04

**Authors:** Dennis Ee See Ong, Yong Meng Hsien, Safinaz Mohd Khialdin, Wan Haslina Wan Abdul Halim

**Affiliations:** 1 Department of Ophthalmology, Universiti Kebangsaan Malaysia Medical Centre, Kuala Lumpur, MYS

**Keywords:** intracranial thrombosis, venous thromboembolism, unilateral abducens nerve palsy, cerebral venous thrombosis, cerebral venous sinus thrombosis

## Abstract

Cerebral venous thrombosis (CVT) is a rare condition characterized by the obstruction of cerebral venous sinuses or cortical veins, leading to stroke-like symptoms. This case report presents a case of a 74-year-old male with isolated unilateral abducens nerve palsy as the sole sign of CVT, without accompanying symptoms or focal deficits. Neuroimaging, including CT and magnetic resonance venography, confirmed the diagnosis of CVT with a thrombus in the right transverse sinus. The patient was co-managed with the medical team and initiated on anticoagulation therapy. Follow-up showed resolution of diplopia and improvement in extraocular muscle movements. Unilateral abducens nerve palsy in CVT is rare, with most cases presenting as bilateral palsy. The case report emphasizes the importance of considering CVT in the differential diagnosis of isolated abducens nerve palsy and highlights the role of neuroimaging in early detection. Timely diagnosis and appropriate management are crucial for favorable outcomes in CVT cases. Further research is needed to enhance understanding of the pathophysiology, prognosis, and optimal management of this uncommon presentation.

## Introduction

Cerebral venous thrombosis (CVT) is an important cause of ischemia in young patients, caused by complete or partial occlusion of the major cerebral venous sinuses (cerebral venous sinus thrombosis, CVST) or the smaller feeding cortical veins (cortical vein thrombosis) [1]. CVST is a relatively rare life-threatening condition with variable symptoms depending on the thrombus location, similar to numerous other neurological disorders [2]. The International Study on Cerebral Vein and Dural Sinus Thrombosis (ISCVT) described the following as the most common presenting symptoms: headache (88.8%), seizures (39.3%), paresis (37.2%), papilledema (28.3%), and mental status changes (22%) [3]. Diagnosis is often delayed to a median period of seven days from the onset of clinical manifestations due to the variability of its symptoms [4]. Global estimation of annual CVST incidence is about three to four cases per million people [2]. It is more common in women than men (2.2:1) because of the sex‐specific risk of thromboembolism [4].

CVST can cause both bilateral and unilateral abducens nerve palsy (cranial nerve (CN) VI palsy), though the frequency of each presentation can vary depending on the underlying cause and location of the venous sinus thrombosis [5,6].

CVST commonly affects the venous drainage of the cavernous sinus, which can lead to impaired blood flow and increased pressure within the sinus. The increased pressure within the cavernous sinus can compress the adjacent sixth cranial nerve, resulting in palsy or paralysis of the nerve. Since the sixth cranial nerve is in close proximity to the cavernous sinus on both sides, bilateral CN VI palsy is more likely to occur when there is a significant disturbance in the venous drainage caused by CVST. However, it is also possible for CVST to cause unilateral CN VI palsy, particularly if the thrombosis is located in a specific area of the venous sinus or if there is an associated intracranial hemorrhage that damages one side of the brain. In these rare cases, the compression or damage to the abducens nerve on one side can lead to unilateral palsy. Local compression typically occurs at the level of the cavernous sinus or the petrous temporal bone [5,6].

It is important to note that bilateral CN VI palsy is a more specific sign of CVST, while unilateral CN VI palsy may be seen in other conditions, such as head trauma or tumors. Other common signs and symptoms of CVST, such as headache, seizures, and focal neurologic deficits, should also be evaluated to arrive at an accurate diagnosis.

This case report was presented previously as a poster presentation at the 2022 6th Asia-Pacific Glaucoma Congress on the 4th of August, 2022.

## Case presentation

A 74-year-old Malay male, a non-smoker with well-controlled diabetes mellitus, presented with horizontal diplopia for three days. There were no other ocular symptoms, such as eye redness, blurred vision, photophobia, or eye pain. He was otherwise well with no associated headache, retro-orbital pain, or other neurological involvement. The remainder of the past medical, surgical, social, family, and medication histories were non-contributory.

He had a best-corrected visual acuity of 6/9 in both eyes (OU), with a near vision of N12. Slit lamp examination showed normal anterior segment findings, normal intraocular pressure (IOP) range, and no relative afferent pupillary defect (RAPD).

**Figure 1:**
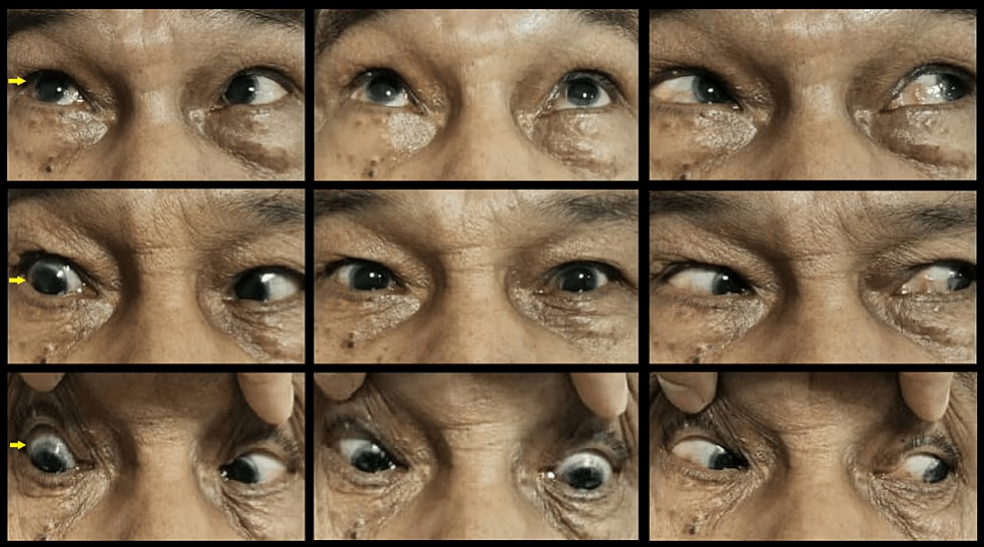
Nine-gaze pictures showing unilateral right lateral rectus under-action secondary to isolated cranial nerve VI palsy.

**Figure 2:**
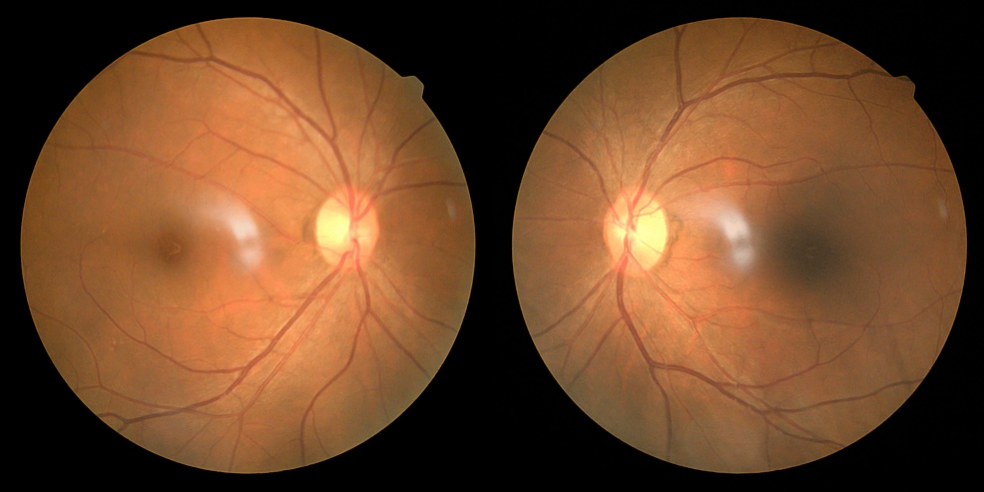
Fundus photo of bilateral eyes showed no optic disc swelling.

A neuro-ophthalmic evaluation revealed binocular horizontal diplopia in dextroversion, with unilateral right (OD) isolated abducens nerve palsy, evidenced by weak abduction in the right eye (Figure 1). Hirschberg test showed normal corneal reflex and no nystagmus was noted. Optic nerve functions were preserved. There was no evidence of optic disc swelling; the retina vessel appeared normal, with good foveal reflex and a normal retina (Figure 2). Spontaneous venous pulsation was present. Other cranial nerves were all intact with no focal neurological deficits. He was afebrile with a temperature of 36.7°C, and other vital signs were within the normal range.

**Figure 3:**
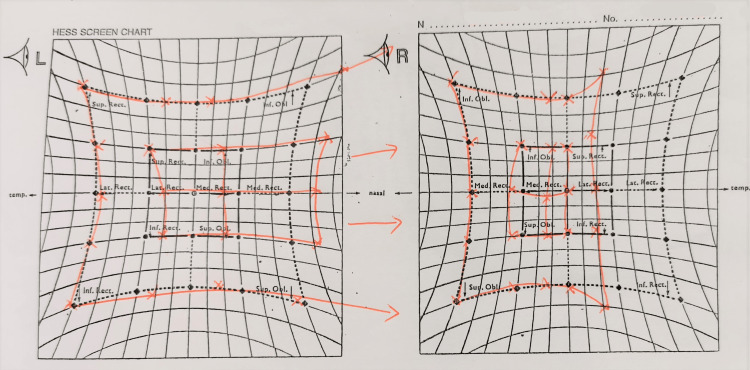
Hess chart showed under-action of the right lateral rectus and over-action of the left medial rectus, suggesting right abducens nerve palsy.

**Figure 4:**
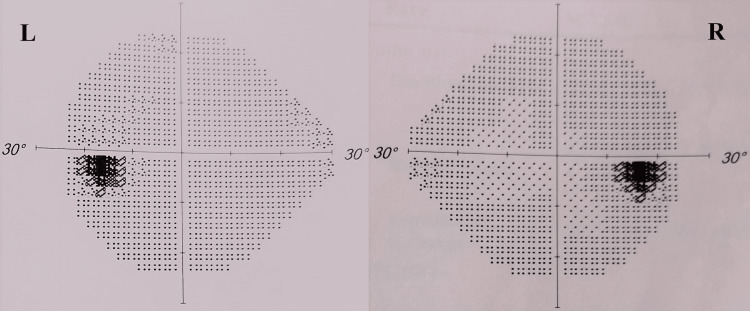
Humphrey visual field test showed no visual defect or any enlarged blind spot.

**Figure 5:**
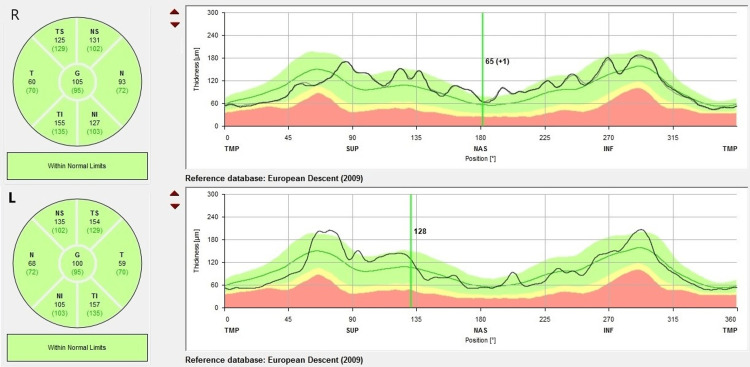
Optical coherence tomography of the retinal nerve fiber layer showed normal thickness with no evidence of optic disc swelling.

Hess chart showed under-action of the right lateral rectus and over-action of the left medial rectus (Figure 3), while no visual defect or enlarged blind spot was seen in Humphrey visual field test (Figure 4). Optical coherence tomography (OCT) of the retinal nerve fiber layer (RNFL) showed normal thickness with no evidence of optic disc swelling (Figure 5).

**Figure 6:**
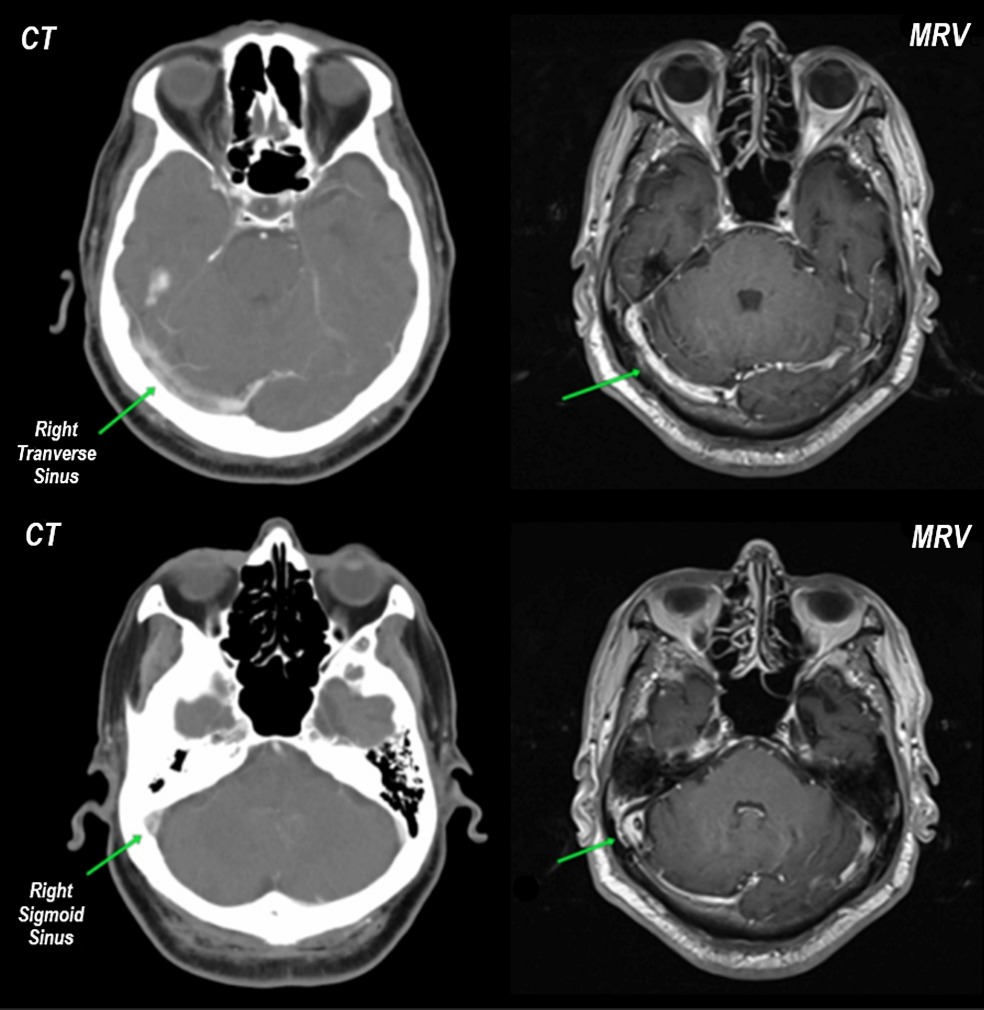
Contrast-enhanced computed tomography (CT) and magnetic resonance venography (MRV) of the brain in a post-gadolinium T1-weighted sequence showed a central linear irregular filling defect of the right transverse sinus extending to the sigmoid sinus. The filling defect is thick and has an irregular outline with peripheral enhancement suggestive of incomplete recanalization of chronic dural venous sinus thrombosis.

Initial computed tomography (CT) and subsequent magnetic resonance venography (MRV) of the brain showed recanalization of the sinus appeared as a linear filling defect of the right transverse sinus extending to the sigmoid sinus and right internal jugular vein with the resolution of edema, suggesting chronic dural venous sinus thrombosis (Figure 6). Otherwise, no focal enhancing brain lesion or enhanced meninges were seen, while bilateral optic nerves and optic chiasm were unremarkable, with normal orbital contents. Infective workup, biohazard, and autoimmune screening showed negative results.

He was then referred to and was co-managed with the medical team for initiation of anticoagulant therapy. Coagulation profiles, including prothrombin time (PT) test, thrombophilia screening, and antiphospholipid screening test, showed negative results. Thrombophilia screening was planned to be repeated after six months; however, it was then declined by the patient. CT of the thorax-abdomen-pelvis was performed to rule out any occult malignancy in view of the hypercoagulable state, and the imaging was unremarkable.

Infective causes were ruled out after a detailed systemic workup. He was diagnosed with unprovoked CVST, supported by strong radiological findings with no identifiable cause or risk factor.

Subcutaneous enoxaparin 60 mg twice daily was commenced for one week, and he was started on oral dabigatran 150 mg twice daily for six months.

**Figure 7:**
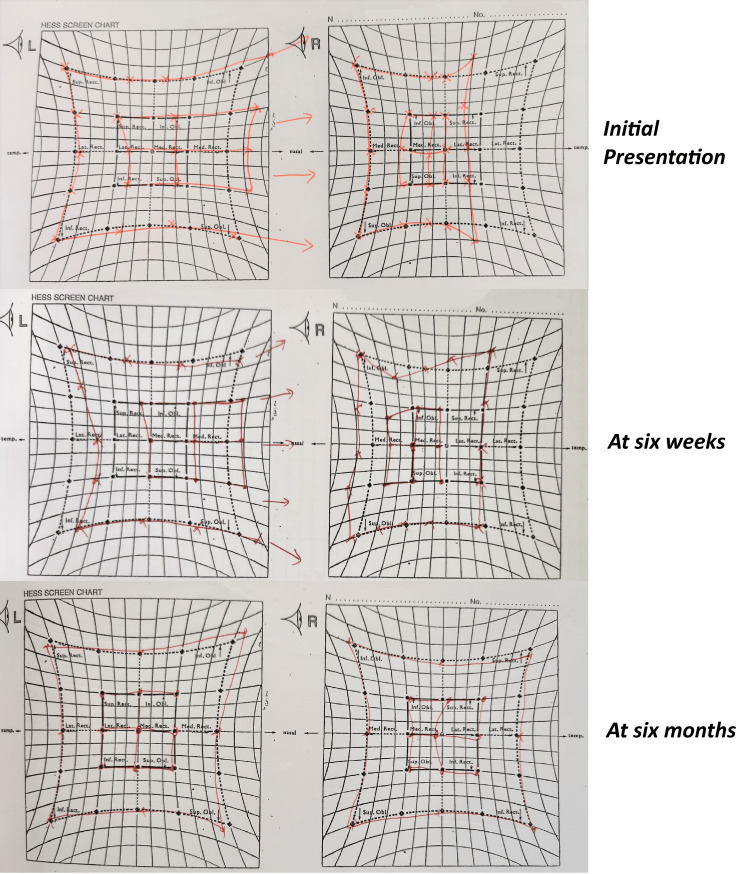
Series of Hess charts showing improvement of under-action right lateral rectus and over-action left medial rectus after six months.

The diplopia was stable after one month and showed resolution during the follow-up after four months, without any new neurological deficit. Extraocular muscle movements normalized, and the repeated Hess chart showed resolved under-action of the right lateral rectus muscle (Figure 7). The visual field remained normal. MRI of the brain was scheduled to assess the recanalization of the sinus. Unfortunately, after his symptoms resolved, the patient defaulted to subsequent follow-up visits and imaging appointments.

## Discussion

Isolated abducens nerve palsy

Isolated abducens nerve palsy refers to a condition characterized by paralysis or weakness of the abducens nerve (CN VI) without the involvement of other cranial nerves. Most isolated abducens nerve palsies are ischemic in nature, but they can also be caused by various other factors, including trauma, infections, inflammation, tumors, and aneurysm [7]. Ischemic damage occurs due to reduced blood supply to the nerve, leading to nerve dysfunction. Reduced blood supply can occur due to common conditions like hypertension, diabetes, and atherosclerosis, while other ischemic causes include vasculitis, migraine, and cranial arteritis. Despite ischemia being the commonest cause of isolated abducens nerve palsy, it is crucial to establish other causes, which require aggressive diagnostic and therapeutic management.

One key factor to consider is the presence of other neurological symptoms and signs. CVST causes sixth nerve palsy and may vary depending on the specific location and extent of the clot. It is possible that the clot directly obstructs the venous drainage of the sixth cranial nerve, leading to ischemia and subsequent nerve dysfunction. Alternatively, the increased intracranial pressure resulting from CVST can indirectly affect the nerve by compressing it against adjacent structures [2]. In CVST, patients often present with additional neurological symptoms such as headache, seizures, altered mental status, and other cranial nerve palsies, whereas patients with isolated CN VI palsy do not have other neurological symptoms.

Additionally, other laboratory tests, such as measures of coagulation and inflammatory markers, may provide further clues to the underlying condition. Imaging studies, such as MRI with venography or CT venography, can also be helpful in identifying CVST as the cause of cranial nerve palsy.

Overall, careful assessment of the patient's clinical presentation, laboratory results, and imaging findings can aid in differentiating the cause of isolated CN VI palsy from CVST [8].

Cavernous venous sinus thrombosis

CVT refers to the formation of blood clots in any of the cerebral venous sinuses. However, CVST specifically refers to clot formation within the cavernous sinus. The cavernous sinus is a complex structure that contains multiple important structures, including cranial nerves, arteries, and veins.

The primary risk factors frequently observed include being in the younger age groups and being female. Additionally, factors such as antibiotic use, dehydration, intracranial tumors, cancer, pregnancy or childbirth, taking oral contraceptives, systemic diseases, thrombophilia, jugular thrombosis after catheterization, fibrous thyroiditis, idiopathic jugular vein stenosis, head trauma, arteriovenous malformations, surgical procedures, and autoimmune disorders are also known to contribute to the risk. At the same time, certain individuals may have multiple risk factors [9].

Thrombosis of the cerebral veins and sinuses is a rare but significant cause of stroke, accounting for 0.5-1.0% of stroke admissions, with an estimated incidence of three to four per million people annually [10]. It is an underdiagnosed condition because of the wide range of signs and symptoms and similarities with many neurological conditions [11].

Towbin (1973) reported a study based on 189 consecutive autopsied cases in three years. A total of 182 brains were examined in these cases with ages ranging from 23 to 95 years, and it was reported that 17 of them (9.3%) were diagnosed with venous thrombosis, suggesting that it might often be missed in life. Among all these 17 cases, they were presented with various symptoms such as lethargy, hemiplegia, dysphagia, paralysis, confusion, and coma [12,13].

Another retrospective study of 181 patients with CVST reported that the most common clinical presentation was headache (47.51%). Other presentations were seizures (24.31%), altered sensorium (14.92%), focal neurological deficit (12.15%), and vertigo (1.1%) [14]. Cranial nerve involvement is generally rare, but bilateral CN VI palsy was typically reported due to elevated intracranial pressure.

In this case, the patient presented with the sole sign of unilateral CN VI palsy without any other symptoms, which was reported once, according to the literature search, where the thrombosis is only localized to the transverse sinus [11]. CVST of idiopathic etiology with isolated abducens nerve palsy as the sole presentation is rare. The unilateral involvement further marks the rarity of this case. Early diagnosis remains a challenge for physicians, as CVST generally has a favorable prognosis if diagnosed and treated early.

The role of neuroimaging has emerged as the best method for the diagnosis of cerebral veins and sinuses thrombosis [8]. Digital subtraction angiography (DSA) has traditionally been regarded as the gold standard for diagnosing CVT. Nowadays, it is primarily employed to guide interventional radiology procedures. The diagnosis of CVT is primarily based on CT, CT venography (CTV), MRI, and MRV. While DSA still surpasses MRV and CTV in terms of providing dynamic information and valuable additional insights, noninvasive neuroimaging techniques with CT and MRI have significantly improved the speed of diagnosis and monitoring, thus influencing the prognosis [15].

Different imaging techniques are commonly employed, such as noninjected or postinjection CT with venous time acquisition and MRI with MRV. CT offers limited information on the clot, vessels, and brain tissue, and provides few indications of intracranial hypertension. However, CTV has demonstrated accuracy in diagnosing cerebral sinus thrombosis. On the other hand, MRI provides comprehensive information on the clot, vessels, brain tissue, and potential signs of intracranial hypertension. Despite being less effective than MRI in detecting brain tissue abnormalities, CT is the preferred initial examination due to its wider availability. It is beneficial for patients who have contraindications for MRI [15].

Treatment is based on the underlying etiology with anticoagulant as the mainstay in non-infectious cases. It should be started as soon as the diagnosis of CVST is confirmed, with rapid anticoagulant therapy, treatment of any underlying cause such as dehydration or sepsis, stopping any prothrombotic medications, control of seizures, and management of intracranial hypertension if required. Patients who deteriorate despite treatment can be considered for endovascular procedures (endovascular thrombolysis or thrombectomy) or neurosurgery (decompressive craniotomy) [1].

Isolated abducens nerve palsy in CVST: a literature review

A literature search revealed five cases of CVST with unilateral CN VI palsy with different etiologies and affected venous sinuses (Table 1).

**Table 1:** Summary of CVST cases reported with unilateral CN VI palsy. CVST: cerebral venous sinus thrombosis; CN: cranial nerve; MRSA: methicillin-resistant Staphylococcus aureus.

Cases	Age	Sex	Thrombosis area	Clinical features (symptoms & signs)	Etiology/risk
Brodsky MC et al. (2018) [16]	15	Female	Superior sagittal sinus and proximal right transverse sinus	Unilateral CN VI palsy, headache, diplopia, bilateral papilledema	Usage of oral contraceptive pills
Tomassini L et al. (2022) [11]	24	Male	Left transverse sinus	Unilateral CN VI palsy, diplopia	Idiopathic
Mittal SO et al. (2017) [17]	31	Male	Inferior petrosal and sigmoid sinus	Unilateral CN VI palsy, neck pain, diplopia, cough	Infection (MRSA bacteremia)
Stam J (2005) [8]	75	Female	Left internal jugular vein	Unilateral CN VI palsy, headache, diplopia	Sclerotic aortic arch
Dorn M et al. (2006) [18]	11	Male	Right sigmoid and transverse sinuses	Unilateral CN VI palsy, headache, diplopia, otalgia	Infection (acute otitis media with mastoiditis)

CVST with unilateral CN VI palsy can occur in a wide range of ages, as shown in Table 1. No specific thrombotic area is responsible for unilateral CN VI palsy. Among the five cases with unilateral CN VI palsy, only one was reported without other symptoms and signs. Headache is the commonest presenting symptom in these five cases, which correlates with the other studies mentioned. No specific etiology or risk factor that is suggestive of direct causative to unilateral CN VI palsy is noted.

## Conclusions

This case report presented a rare case of isolated unilateral abducens nerve palsy as the sole sign of CVT, emphasizing the importance of considering CVT in the differential diagnosis of isolated cranial nerve palsies. The case highlighted the significance of neuroimaging, such as CT and MRV, in confirming the diagnosis of CVT with a thrombus in the right transverse sinus. The patient's favorable outcome following early initiation of anticoagulation therapy underscored the importance of timely diagnosis and appropriate management in CVT cases. This case report serves as a reminder for healthcare professionals to be vigilant and consider CVT as a potential cause when evaluating patients with isolated cranial nerve palsies. Neuroimaging may be necessary in cases of CN VI palsy with atypical features, recurrent palsy, persistent or worsening symptoms, associated neurological deficits, and significant risk factors. Further research is needed to enhance our understanding of the pathophysiology, prognosis, and optimal management of this rare presentation. By increasing awareness and knowledge, healthcare providers can improve the recognition and outcomes of CVT cases with atypical manifestations.
